# Editorial: Climate is changing: harnessing plant-microbe interactions for sustainable agriculture in arid areas

**DOI:** 10.3389/fmicb.2025.1687842

**Published:** 2025-10-14

**Authors:** Camilla Fagorzi, Erna Karalija, Agnese Bellabarba

**Affiliations:** ^1^Department of Biology, University of Florence, Florence, Italy; ^2^Genexpress Laboratory, Department of Agronomy, Food, Environmental and Forestry Sciences (DAGRI), University of Florence, Florence, Italy

**Keywords:** climate change, plant-microbe, sustainable agricultural applications, bacterial inocula, biopriming

**Figure 1:**
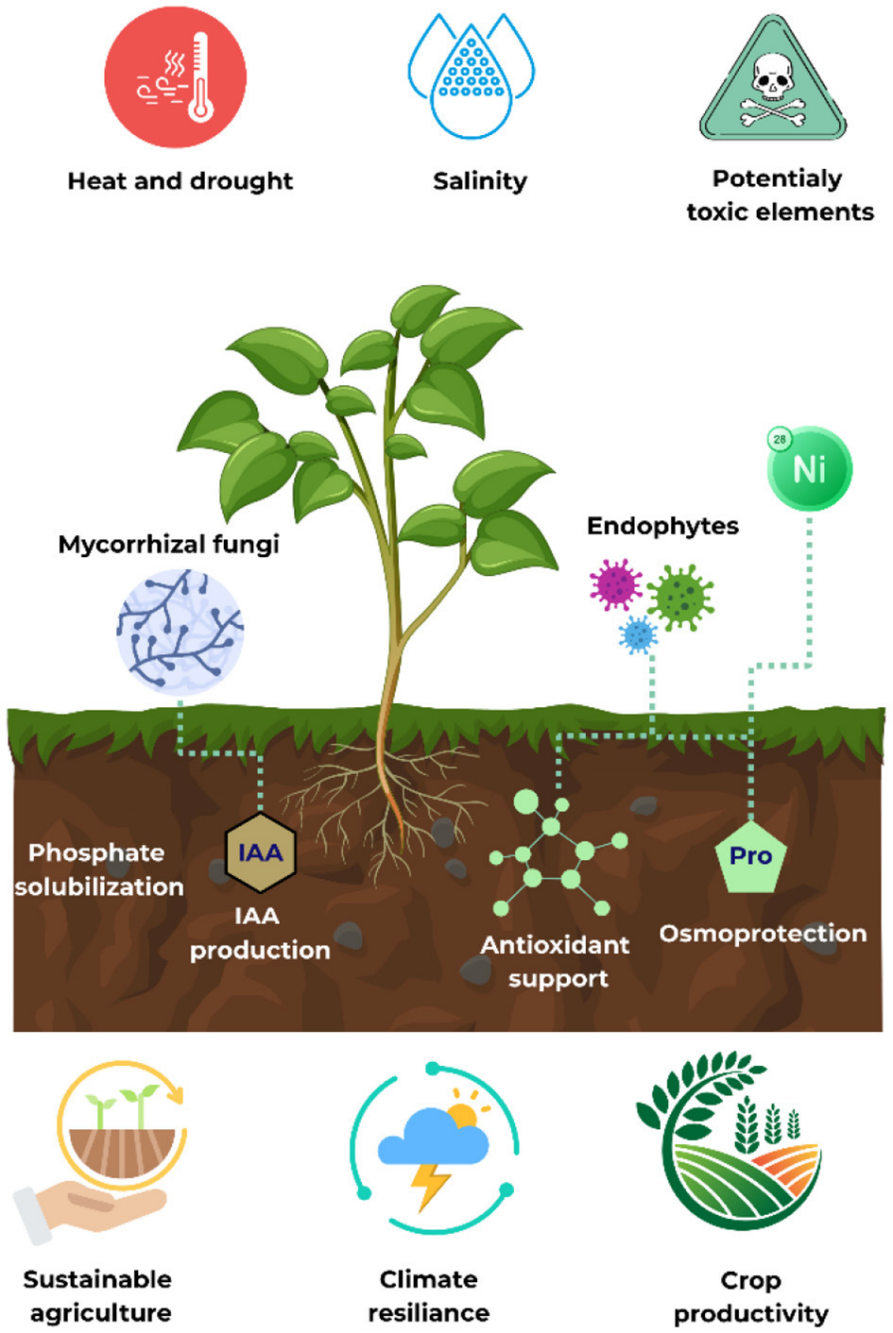
Elements that play a fundamental role in strategies to improve plant stress resilience for the development of sustainable crop system in a climate-changing context.

Climate change, land degradation, water scarcity, and soil contamination are increasingly undermining agricultural productivity, especially in arid and semi-arid regions. Traditional farming approaches are no longer sufficient to ensure long-term food security or ecological resilience. In this context, the use of plant-associated microbiomes has emerged as a promising strategy to improve plant stress resilience, reduce dependence on chemical inputs, and foster sustainable crop systems (Figure 1).

This Research Topic brings together studies that showcase the diversity and applied potential of microbiome-based strategies for enhancing crop tolerance to environmental challenges such as drought, nutrient limitations, and heavy metal contamination. A recurring theme throughout the contributions is the shift away from generic bioinoculant solutions toward tailored, system-specific applications, reflecting the growing awareness that microbial interventions must be ecologically and agronomically contextualized.

For instance, one presented study (Hone et al.) investigated seed microbiomes from commercial *Medicago sativa* cultivars, isolating novel phosphate-solubilizing strains and revealing a disconnect between genomic markers and actual plant performance. This emphasizes the need for in planta validation and multi-layered screening to enhance predictive accuracy in biofertilizer development. Another contribution of Peng et al. explored halophyte-associated microbiota, identifying two novel strains from *Salicornia europaea* roots with high salinity tolerance and growth-promoting traits. These extremes tolerant microbes expand the toolbox for addressing the growing salinization of agricultural soils. Microbial–plant–signal interactions are also examined in this Research Topic. One study on *Camellia oleifera* demonstrated how glutamate supplementation reshapes the rhizosphere bacterial community under drought, enhancing nitrogen cycling and root–microbe synergy (Lu et al.). This points to amino acid-mediated modulation as a new frontier in designing biostimulants that act on both plant and microbial targets. Heavy metal stress, an overlooked yet critical issue in many arid zones, is addressed through a study on *Solanum lycopersicum* bioprimed with *Paraburkholderia phytofirmans* PsJN (Hasanović et al.). This work documents physiological improvements and biochemical resilience in plants exposed to nickel stress, reinforcing the translational potential of well-characterized PGPR in contaminated soils. The studies included in this Topic illustrate a move toward “climate-smart” microbiome solutions. From seed microbiota to signaling pathways and field-level biopriming, they highlight the importance of ecological fit, microbial functionality, and experimental validation. This Research Topic calls for a new generation of microbial technologies that are not only resilient and adaptive but also tailored to local agroecological realities. We thank all contributing authors and reviewers for advancing this vibrant field of research. We also acknowledge the *Frontiers in Microbiology* editorial team for their guidance throughout the process. We hope this Research Topic inspires future innovations at the intersection of microbiome science and sustainable agriculture.

